# Frozen thawed embryo transfer cycles; A comparison of pregnancy outcomes with and without prior pituitary suppression by GnRH agonists: An RCT

**Published:** 2018-09

**Authors:** Alamtaj Samsami, Zohre Chitsazi, Golnaz Namazi

**Affiliations:** *Department of Obstetrics and Gynecology, Shiraz University of Medical Sciences, Shiraz, Iran.*

**Keywords:** Embryo transfers, In vitro fertilization, Ovarian stimulation, Gonadotropin releasing hormone, Agonist

## Abstract

**Background:**

To perform an in-vitro fertilization cycle, pretreatment with gonadotropin-releasing hormone (GnRH) agonist is widely used as a part of controlled ovarian hyper-stimulation protocols to prevent endogenous luteinizing hormone surge and spontaneous ovulation. GnRH agonist pretreatment is relatively costly and there is a risk of hypo estrogenic side effect. It would also lengthen the preparation period until pituitary desensitization occurs.

**Objective:**

Our study is aimed at evaluating the pregnancy outcome rate of frozen thawed embryo transfer with and without GnRH agonists pretreatment.

**Materials and Methods:**

Women with documented infertility who were candidate for frozen thawed embryo transfer were recruited and randomly assigned to two groups. In group A (n=100), patients received GnRH agonist, Buserelin, to induce pituitary desensitization prior to endometrial preparation and embryo transfer. Individuals in group B (n=100) received steroid manipulation without prior down-regulation of the pituitary. Chemical pregnancy, implantation rate, clinical pregnancy and ongoing pregnancy were measured and statistically compared between the two groups.

**Results:**

None of the outcome measures including clinical and chemical pregnancy rates, implantation rate, and ongoing pregnancy rate showed significant difference between the two groups. Similarly, the rate of miscarriage did not vary between the two groups.

**Conclusion:**

In this study, we found that removing the GnRH agonists pretreatment from the programmed cycles did not negatively influence the pregnancy outcome or implantation rate. Moreover, it will cause a considerable reduction in cost of assisted reproductive technology as well as adverse effects related to GnRH agonists, while having a favorable implantation and pregnancy outcomes.

## Introduction

Infertility as an increasingly common reproductive health issue is estimated to affect approximately 10-15% of couples in industrialized countries (1). In-vitro fertilization (IVF) is one of the most successful methods for assisted reproductive technologies (ART) usually used as the last resort for both male and female factor infertilities. Recently, implantation of fresh embryos has been replaced by cryopreservation of multiple embryos for later implantation at multiple occasions to reduce the rate of failure. This latter approach is called Frozen-Thawed Embryo Transfer (FTET) (2). 

The first step of IVF procedure which is consisted of ovulation induction can be performed in either spontaneous cycles or hormone replacement cycles. In hormone replacement cycles, ovaries are stimulated to release several eggs in a cycle. Introduction of controlled ovarian hyperstimulation (COH) has contributed to significant increase in IVF success rate in the recent years. Various protocols of COH are employed by institutions and clinics based on their own expertise and experience. Current COH protocols pursue three general objectives: 1) pituitary suppression, 2) multiple follicle growth stimulation, and 3) ovulation induction. Pituitary suppression is achieved by administration of gonadotropin-releasing hormone (GnRH agonist with the aim of preventing premature luteinizing hormone surge and thus decreasing cycle cancelation (3).

Hormone replacement cycle (aka artificial cycle) can be favorable in older patients and those with irregular menstrual cycles/ovulation as it allows for easy management, flexibility in timing of the frozen mmbryo transfer (4-6) and has a lower cancellation rate. With FTET being performed, hormone replacement cycles are induced for synchronization of endometrium and embryo development (6). 

Nowadays, most of the institutions induce pituitary suppression in the first step of COH. This can be achieved by administration of a GnRH agonist. The aim of pretreatment with GnRH agonist is to avoid spontaneous luteinizing hormone surge and subsequent ovulation. However, this protocol is costly and there is a risk of hypoestrogenic side effect that would lengthen the preparation. Recent studies have compared protocols with and without GnRH agonist pretreatment and reported similar clinical outcomes (7, 8). However, GnRH agonists are still actively administered in ART protocols. Clearly, the program with an absence of GnRH agonist may be preferred because of its simplicity and low cost. 

Considering the inconsistencies in current literature about using GnRH agonists in artificial cycles in terms of pregnancy outcomes, this experiment was designed as a randomized controlled trial to investigate several aspects of clinical outcome of IVF-FTET protocol without GnRH agonist pretreatment, compared to the protocol with GnRH agonist trial in a population of southern Iranian women with documented infertility.

## Materials and methods

**Subjects**

All individuals with established medical diagnosis of infertility who referred to Shahid Faghighi hospital, affiliated to Shiraz University of Medical Sciences, Shiraz, Iran for IVF from November 2014 to November 2015 invited to participate in the study. 

Inclusion criteria for this study were as follows:

- history of infertility
- 20< age< 39 yr
- Couples undergoing ART with their own gametes
- Women stimulated with a long GnRH agonist protocol of IVF (for group A)
- Couples having frozen embryo available for transfer

Subjects with the following criteria were excluded from the study

- Age >39 yr
- High-grade endometriosis
- Existence of myoma or adhesion in uterus
- Body mass index more than 29 or less than 18 kg/m^2^
- Oocyte donation cycles

**Intervention**

A total of 237 women were randomly allocated in two groups (119 and 118 cases in groups A and B, respectively) using computerized randomization codes. Neither patients nor project executives were blinded of patients’ allocations. In group A (control) patients received a trial of GnRH agonist prior to ovulation induction, while individuals in group B (case group) only received steroids without prior down-regulation of the pituitary using GnRH agonist. In group A, 0.5 mg of Buserelin acetate (a GnRH agonist) (Suprefact, Hoechst AG, Germany) was injected subcutaneously daily starting on the 21^st^ day of menstrual cycle (mid-luteal phase). On the first day of menstruation, GnRH agonist dose was reduced to 0.3 mg/day subcutaneous. Participants in group B did not receive GnRH agonist for pituitary down-regulation. 

In both groups, Estradiol Valerate (Abureyhan, Iran) was administered orally, starting on the second day of the target cycle with a dosage of 6 mg/day for endometrial preparation. In the morning of cycle day 8-9 (6-7 days following Estradiol initiation), an ultrasound examination was performed for all of the participants to assess endometrial thickness, and pattern. Using a 5 MHz vaginal transducer, the uterus was visualized longitudinally. 

Endometrial thickness was measured and recorded as the maximum distance (mm) between myometrium and endometrial surface. A thickness of ≥8 mm was considered satisfactory for initiation of embryo transfer. Estradiol dosage was increased to 8 or 10 mg/day for woman who failed to achieve desired endometrial thickness. 

For each participants, follow-up ultrasound examinations were performed at 3-day intervals until achieving satisfactory endometrial thickness. Prior to embryo transfer, individuals in both groups received progesterone in oil (prontogest, Amsa, Rome, Italy) 100 mg/day (IM) starting on the day which desired endometrial thickness was achieved. In group A, Suprefact was discontinued at this point and progesterone was administered for 3 consecutive days.

Thawed embryos were transferred on the 3^rd^ day following progesterone administration using labotect catheters (Labor technik, Germany) which was introduced to the uterus via the cervix under ultrasound guide and desired location for transfer of embryos was detected. Embryos were transferred to a point of the uterus at 1.5 cm distance from fundus. All embryo transfers were performed by one gynecologist physician, an expert in infertility treatments and an associate professor of gynecology. Patients were monitored for 3 hr in the recovery room after the procedure was accomplished.

Both estradiol and progesterone were administered until the 12^th^ wk of gestation. Individuals with serum β Human chorionic gonadotropin (βHCG) level >10 IU/L checked 16 days following thawed embryo transfer were confirmed to have a chemical pregnancy. Another serum βHCG measurement was conducted to observe satisfactory rising levels. In woman with negative chemical pregnancy, medications were discontinued. Ultrasound examination were performed 5 wk from the date of embryo transfer for all participants.

**Cryopreservation**

All participants were IVF candidates who had their own pre-embryos cryo-preserved during the previous cycle (the cycle preceding embryo transfer). The procedure of cryopreservation was conducted at 8-cell stage using 1, 2- propanediol and sucrose as the cryo-protectants. Freezing and thawing solutions consisted of the cryo-protectants in a PBS plus HEPES medium with 20% W/V human serum albumin. Cryopreservation of all embryos was undertaken with vitrification in both groups. 

After two-step loading with equilibration solution that contained ethylene glycol and dimethyl sulfoxide and a vitrification solution that contained ethylene glycol, dimethyl sulfoxide, and sucrose, a narrow glass capillary was used to load the embryos onto the cryo-top. After loading, the majority of the solution was removed to leave only a thin layer that covered the embryos, after which the sample was quickly immersed into liquid nitrogen. Subsequently, the plastic cap was pulled over the film part of the cryotop and the sample stored in liquid nitrogen. Thawing was performed by the transfer of cryo-tubes into a warm bath at a temperature of 35^o^C. After complete thawing, embryos were taken through a series of decreasing concentrations of propanediol and sucrose, washed three times in PBS and placed in a fresh equilibrated, warmed culture medium. Embryos that survived the freezing/thawing procedure (50% of their initial number of blastomeres intact) were transferred to the recipient uterus within 30 min.

**Embryo grading**

Before freezing, embryos were examined and classified as three types according to blastomere symmetry and the relative proportion of anucleate fragments in the zona pellucida:

- Type A: Regular blastomeres with no fragmentation,
- Type B: Irregular and asymmetrical blastomeres but no fragmentation,
- Type C: Regular or irregular blastomeres with 20% of their content filled with anucleated fragments.

Embryos with 50% of their surface filled with anucleated fragments were not eligible for freezing or transfer and were discarded. 

**Outcome measures**

- Serum βHCG level >10 IU/L obtained 16 days following thawed embryo transfer, was defined as chemical pregnancy.
- Clinical pregnancy was established by the observation of intrauterine gestational sac daring ultrasound examination 5 wk after transfer date.
- Ongoing pregnancy was defined as the presence of intrauterine fetal heart motion 12 wk after transfer date on ultrasound examination.
- The implantation rate was defined as the number of gestational sacs with fetal pole detected using vaginal ultrasound to the number of transferred embryos.
- Miscarriage rate was defined as fetal loss under the 20^th^ wk of gestation.

**Ethical consideration**

This study was conducted as a randomized controlled trial and approved by the Ethics Committee of Shiraz University of Medical Sciences (#2018-363). Study design, risks and benefits were explained and written informed consent was obtained from those willing to participate.

**Statistical analysis**

All statistical analyses were performed with the statistical Package for Social Sciences version 17.0 (SPSS Inc., Chicago, IL, USA). The significance of differences between two groups was assessed using independent T-tests (comparison of patient demographics between groups), Chi square test (comparison of pregnancy outcome between groups), Fisher’s exact test (comparison of miscarriage rate between groups) and Wilcoxon-Mann-Whitney test (comparison of mean implantation rate between groups). A two-sided p<0.05 was considered statistically significant. 

Sample size for this study was determined using G-power software with power=80% and α=0.05. Data are presented as mean± SD.

## Results

**Descriptive findings**

**Figure 1:**
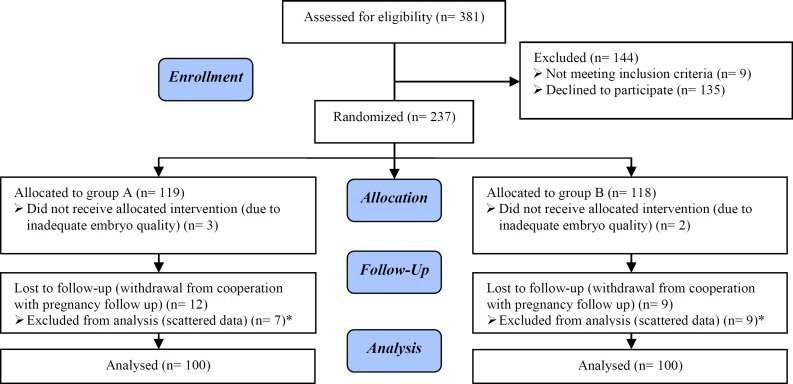
CONSORT flowchart for recruitment of candidates

A total number of 381 women who were scheduled for IVF were invited to participate in the study. 237 patients agreed and signed an informed consent to enter the project. These woman were randomized in two groups, 119 women in group A and 118 women in group B. 3 patients from group A and 2 patients from group B were dropped from the study because of inadequate quality of the embryo. 12 cases from group A and 9 cases from B left the study due to inability to attend all the appointments regularly. From the remaining 109 and 107 individuals in groups A and B respectively, data from 100 patients in each group were finally used for statistical analysis to provide the best matching between the two groups regarding the baseline parameters. Figure 1 demonstrates the consort flowchart for recruitment of study population. 

The study population had a mean age of 30.4±5.13 yr. The mean age of participants in group A was 30.76±5.51 comparing to 30.14±4.76 in group B, with no statistically significant difference (p=0.381). Baseline characteristics of the studied individuals are represented in table I. Individuals in both groups were matched regarding age, primary endometrial thickness, number of days of cycle preparation with progesterone prior to embryo transfer, maximum and starting doses of administered estradiol.

**Achievement of pregnancy**

Ongoing pregnancy until 12^th^ wk of gestation was achieved in 18 cases in group A and 16 patients in group B (totally 36 patients comprising 18% of the study population). Statistical comparison of this outcome between the two groups was made using Chi-Square test rejected the hypothesis that there is a significant variation between the two groups (p=0.677). 

**Miscarriage**

Overall, 5 individuals (three in group A and two in group B) had lost their fetuses before the 20^th^ wk of gestation who were marked to have pregnancy loss (miscarriage). Considering the small number of mothers with miscarriage, a Fisher’s Exact Test was conducted to calculate possible differences between the study groups. Statistically, this variation between the two groups was found insignificant (p=0.518). 

**Implantation rate**

Individuals in group A had a mean implantation rate of 0.08±0.17, while the value was 0.06±0.17 in patients in group B. There was no statistically significant difference between the study groups in terms of implantation rate (p=0.512). In other words, the hypothesis that GnRH agonist treatment affects the rate of implantation was rejected.

**Embryo grading**

Individuals allocated in treatment group had received a mean of 1.7±1.2 embryos graded as A and subjects in group B had received a mean of 1.6±1.2 grade A embryos with no statistically significant difference (p=0.48). For Embryos graded as B, patients in the GnRH agonist treated group had a mean of 0.8±0.9 embryos while the value was 0.9±1.1 in the group without GnRH treatment. No statistically significant difference was noted (p=0.39). Both study groups had received a similar number of embryos graded as C. An average number of 0.25 grade C embryos was transferred in GnRH agonist treated group while the value was 0.16 in the group not treated with GnRH agonist (p=0.32). Overall both groups were matched regarding the grading of embryos transferred.

**Table I:** Independent T-test comparison of baseline characteristics of the studied patients

	**Group A**	**Group B**	**p-value**
Number of participants	100	100	
Age (yr)	30.76 ± 5.55	30.14 ± 4.76	0.381
Primary endometrial thickness (mm)	4.5 ± 0.71	4.4 ± 0.63	0.235
Endometrial thickness at the start of progesterone (mm)	8.14	8.00	0.183
Number of days of cycle preparation	15.26 ± 3.04	15.42 ± 2.63	0.676
Max estradiol dose (mg)	7.5 ± 1.47	8.1 ± 1.68	0.003

Data presented as mean±SD.

Independent *t*-test

**Table II:** Chi square comparison of pregnancy outcome and comparison between groups

	**Total**	**Group A**	**Group B**	**p-value**
Chemical pregnancy (16 days after embryo transfer)	34 (15.7%)	18 (16.5%)	16 (14.9%)	0.677
Clinical pregnancy (positive gestational sac and embryo visualization 6 weeks after embryo transfer)	34 (15.7%)	18 (16.5%)	16 (14.9%)	0.677
Ongoing pregnancy (maintaining pregnancy until 12 week gestational age)	34 (15.7%)	18 (16.5%)	16 (14.9%)	0.677
Miscarriage (pregnancy loss after 12 weeks up to 20 weeks of gestational age)	5 (2.5%)	3 (2.7%)	2 (1.8%)	0.518
Mean Implantation rate		0.08 ± 0.17	0.06 ± 0.17	0.512

Data presented as frequency (%) per group.

Chi square test/Man Mann–Whitney U test for mean implantation rate

*Patients whose data were withdrawn from analysis had incomplete records such as serum βHCG levels and IVF protocol details.

## Discussion

The present study was conducted as a prospective randomized controlled trial with the aim of comparing results of frozen thawed embryo transfer cycles in an artificially prepared endometrium with and without prior use of GnRH agonist for pituitary suppression in a population of southern Iranian women. To our knowledge, only one study with similar goals was conducted in Iran. As indicated in the section on results, there was no statistically significant difference between the study groups in terms of pregnancy outcomes. Neither of study groups showed higher success rate for chemical, clinical, ongoing pregnancy and miscarriage rates as the primary measures of outcome. Our results indicate that transfer of frozen thawed embryos could be successfully performed in a programmed cycle without the use of GnRH agonist prior to endometrial preparation.

There are other studies with similar methodologies published in the literature yielding similar results. In a prospective randomized trial conducted by Davar and colleagues, 60 patients were randomly divided into two treatment groups (9). Group A (n=30) commenced steroid supplementation without prior pituitary desensitization; whereas group B (n=30) had pituitary suppression using Buserelin acetate (0.5 mg SC), prior to steroid hormone administration (9). The implantation rate, chemical and clinical pregnancy rates were 1.6, 10 and 6.6% in the group A and 3, 13.3 and 10% in the group B. Although chemical and clinical pregnancy rates were lower in Davar’s study comparing to our results (17 and 17.5% respectively), their findings indicated that there was no significant difference in implantation and pregnancy rates between both groups. It should be mentioned that relatively small number of studied subjects in Davar’s study might serve as a limiting factor in discussing their findings.

In another prospective randomized study conducted by Simon and co-worker, the possibility that the programmed cycle for embryo transfer could still be hormonally manipulated without the use of GnRH agonist was studied (8). They compared the outcome of frozen thawed embryo transfer cycles using micronized 17â-estradiol and micronized progesterone preparations with and without the concomitant use of GnRH agonist. Reported pregnancy rate per embryo transfer and implantation rate in group A (26.4% and 9.5%) were comparable to those of group B (21.1% and 9%). In both our study and Simon’s experiment, the two study groups were similar in their profile including age, embryo grading before cryopreservation, endometrial thickness on the day of progesterone administration and number of days of cycle preparation. However, the GnRH agonist agent used in Simon’s study was Decapeptyl in single dose IM form (8). Their reported implantation rate was higher compared to our findings (9.5% and 8% respectively) which could be attributed to the GnRH agonist agent used in their experiment (8). Considering the similar reported results here, it is concluded that compared to GnRH agonist programmed cycles, cycles without GnRH agonist pretreatment are more convenient for both the patient and medical staff and results in a similar success rate at a lower cost. Moreover, patients will have the option of choosing the desired cycle for embryo transfer and are considerably more comfortable regarding medication use. The direct medical cost of the frozen embryo transfer (FRET) cycle will be reduced resulting in patients’ satisfaction especially in developing countries such as Iran.

In a study of 162 patients with a total of 199 thawed embryo transfer cycles, conducted by Queenan and colleagues, Patients achieved Endometrial preparation in a programmed cycle utilizing exogenous hormone without GnRH agonist pretreatment. Their results showed an ongoing pregnancy rate of 16.4% which lies in agreement with our findings (17%) (7).

However, the overall implantation rate reported in Queenan’s study was 6 % which was lower compared to a value of 8% in our study. A possible explanation of this inconsistency could be the age of participants in Queenan’s study (35±4 yr) which was more advanced compared to a value of (30±0.4 yr) in our experiment (7). As extensively studied in the literature, patients’ advanced age, negatively influences the outcome of cryopreservation embryo transfer (10). The overall pregnancy rate (chemical) was 29.2 % in Queenan’s study which was comparably higher to 17.5% reported here. It could be explained by the fact that individuals in our study had a mean of 8 mm endometrial thickness, while the value was 10.8±2 mm in Queenan’s experiment. There are conflicting studies in the literature around the effect of endometrial thickness on pregnancy outcome in ART; however, it has been documented that an improved pregnancy rate is associated with a thicker endometrium (11). 

Moreover, higher pregnancy rates among donor oocyte recipients were reported with an endometrium that was >10 mm thick (9 vs. 38.7%; p<0.01) (12). Finally, it should be mentioned that endometrial pattern may serve as a better predictor of pregnancy outcome comparing to endometrial thickness because several authors have described higher pregnancy rates in cycles displaying the triple line pattern versus the hyperechoic pattern (7). It is suggested that in future studies, a possible relation between endometrial ultrasound patterns and pregnancy outcome could be evaluated.

## Conclusion

In this study, we presented combined favorable aspects of the natural cycle and the GnRH agonist programmed cycles. We believe this modification of programmed cycles with omitting GnRH agonists will result in reduction of many previously experienced adverse effects including risk of ovarian cyst formation and hypoestrogenic side effects. Moreover, it will cause a considerable reduction in cost of ART in our country with convincing pregnancy and implantation outcomes. We have indicated that hormonal supplementation without prior GnRH a down-regulation does not adversely affect pregnancy or implantation. Pregnancy and implantation rates in the present study are similar to those reported in other studies around the world. 

As compared to GnRH agonist programmed cycles, unprepared cycles are much simpler, more convenient for both the patient and the medical staff, and results in a similar success rate but with lower cost. It is recommended that long-term pregnancy outcomes including live birth rate, prenatal and obstetric outcomes of unprepared cycles should be studied in our country to provide true insight to the economics and cost-benefits of assisted reproductive treatments. 

## Note

***Registration ID in RCT: IRCT2017052834184N1***

***This article extracted from M.D. thesis. (Zohre Chitsazi)***

## References

[1] Boivin J, Bunting L, Collins JA, Nygren KG (2007). International estimates of infertility prevalence and treatment-seeking: potential need and demand for infertility medical care. Hum Reprod.

[2] Wang J, Sauer MV (2006). In vitro fertilization (IVF): a review of 3 decades of clinical innovation and technological advancement. Ther Clin Risk Manag.

[3] Pacchiarotti A, Selman H, Valeri C, Napoletano S, Sbracia M, Antonini G (2016). Ovarian stimulation protocol in IVF: An up-to-date review of the literature. Curr Pharm Biotechnol.

[4] Bals-Pratsch M, Al-Hasani S, Schopper B, Diedrich C, Hoepfner AS, Weiss J (1999). A simple, inexpensive and effective artificial cycle with exogenous transdermal oestradiol and vaginal progesterone for the transfer of cryopreserved pronucleated human oocytes in women with normal cycles. Hum Reprod.

[5] Ben-Nun I, Shulman A (1997). Induction of artificial endometrial cycles with s oestrogen implants and injectable progesterone in in-vitro fertilization treatment with donated oocytes: a preliminary report. Hum Reprod.

[6] Edgar DH, Gook DA (2012). A critical appraisal of cryopreservation (slow cooling versus vitrification) of human oocytes and embryos. Hum Reprod Update.

[7] Queenan J, Ramey JW, Seltman HJ, Eure L, Veeck LL, Muasher SJ (1997). Transfer of cryopreserved-thawed pre-embryos in a cycle using exogenous steroids without prior gonadotrophin-releasing hormone agonist suppression yields favourable pregnancy results. Hum Reprod.

[8] Simon A, Hurwitz A, Zentner BS, Bdolah Y, Laufer N (1998). Transfer of frozen-thawed embryos in artificially prepared cycles with and without prior gonadotrophin-releasing hormone agonist suppression: a prospective randomized study. Hum Reprod.

[9] Davar R, Eftekhar M, Tayebi N (2007). Transfer of cryopreserved-thawed embryos in a cycle using exogenous steroids with or without prior gonadotropihin-releasing hormone agonist. J Med Sci.

[10] Kolibianakis EM, Zikopoulos K, Devroey P (2003). Implantation potential and clinical impact of cryopreservation-a review. Placenta.

[11] Kovacs P, Matyas S, Boda K, Kaali SG (2003). The effect of endometrial thickness on IVF/ICSI outcome. Hum Reprod.

[12] Check JH, Nowroozi K, Choe J, Lurie D, Dietterich C (1993). The effect of endometrial thickness and echo pattern on in vitro fertilization outcome in donor oocyte-embryo transfer cycle. Fertil Steril.

